# An explicit immunogenetic model of gastrointestinal nematode infection in sheep

**DOI:** 10.1098/rsif.2014.0416

**Published:** 2014-10-06

**Authors:** Joaquín Prada Jiménez de Cisneros, Michael J. Stear, Colette Mair, Darran Singleton, Thorsten Stefan, Abigail Stear, Glenn Marion, Louise Matthews

**Affiliations:** 1 Institute of Biodiversity, Animal Health and Comparative Medicine, University of Glasgow, Garscube Campus, Bearsden Road, Glasgow G61 1QH, UK; 2 Boyd Orr Centre for Population and Ecosystem Health, University of Glasgow, Glasgow G61 1QH, UK; 3 Biomathematics and Statistics Scotland, The King's Building, Edinburgh EH9 3JZ, UK

**Keywords:** host–parasite model, approximate Bayesian computation, helminth infections, selective breeding, sheep, nematodes

## Abstract

Gastrointestinal nematodes are a global cause of disease and death in humans, wildlife and livestock. Livestock infection has historically been controlled with anthelmintic drugs, but the development of resistance means that alternative controls are needed. The most promising alternatives are vaccination, nutritional supplementation and selective breeding, all of which act by enhancing the immune response. Currently, control planning is hampered by reliance on the faecal egg count (FEC), which suffers from low accuracy and a nonlinear and indirect relationship with infection intensity and host immune responses. We address this gap by using extensive parasitological, immunological and genetic data on the sheep–*Teladorsagia circumcincta* interaction to create an immunologically explicit model of infection dynamics in a sheep flock that links host genetic variation with variation in the two key immune responses to predict the observed parasitological measures. Using our model, we show that the immune responses are highly heritable and by comparing selective breeding based on low FECs versus high plasma IgA responses, we show that the immune markers are a much improved measure of host resistance. In summary, we have created a model of host–parasite infections that explicitly captures the development of the adaptive immune response and show that by integrating genetic, immunological and parasitological understanding we can identify new immune-based markers for diagnosis and control.

## 1. Introduction

Gastrointestinal nematode infection is arguably the major disease affecting small ruminants [1,2]. Different nematodes cause different pathologies. In cool temperate climates such as the UK, the predominant nematode in sheep is *Teladorsagia circumcincta* and this causes a relative protein deficiency [3] which affects growth and production and in extreme cases can kill the host. Economically efficient and welfare friendly sheep husbandry therefore requires the control of these parasites. Historically, nematode infections have been controlled at least partly by anthelmintic treatment, but the evolution of resistance to drug treatment [4,5] means that alternative methods of parasite control are urgently needed.

Mathematical models have been extensively used to gain insights into the dynamics of host–parasite interactions in humans, wildlife and livestock, and to help identify effective control measures [6–9]. Since the review by Smith & Grenfell [10], the dynamics of gastrointestinal parasites of ruminants have received considerable modelling attention. The models developed, which have been reviewed elsewhere [11,12], range in complexity from relatively simple phenomenological models [13–15] to detailed models that capture the multiple stages of the parasite life cycle within and outwith the host, allowing effects such as temperature, climate, grazing behaviour, nutrition and management to be incorporated [10,16–19]. The focus of these models has encompassed the use of grazing management as a control measure [20,21], the impact of drenching regimes [16], the generation and spread of anthelmintic resistance [18,22–26], selective breeding for disease resistance [27] and the implementation of targeted or strategic treatments [28], including the unexpected prediction that estimated breeding values for faecal egg counts (FECs) derived from pedigrees were less effective tools than the original data [29].

Two important themes that recur in these modelling studies are the aggregation of infection loads and the acquisition of immunity by the host. Some studies have characterized the observed aggregation of parasite burdens across numerous host–parasite systems [30–33], whereas mathematical modelling has been used to investigate the mechanisms and consequences of aggregation [30,34–38]. The studies by Cornell *et al.* [39] and Grenfell *et al.* [35] suggest that much of the observed variation in parasite burden between hosts is attributable to some form of host heterogeneity.

Although the importance of acquired immunity has long been recognized, there are few models addressing in detail the ‘immunoepidemiology’ of farmed ruminants. Host immunity has been assumed to increase over time following exposure to infective larvae, and to reduce the establishment, fecundity and survival of adult parasites [14,40]. However, Roberts [41] identifies a need to move beyond the common phenomenological approaches to host immunity in host–nematode models in order to facilitate the integration of epidemiological models with data from immunological studies. Hellriegel [42] and Stear *et al.* [43] also issued calls for the greater integration of immunology, parasitology, genetics, epidemiology, mathematical modelling and statistics in host–parasite models.

*Teladorsagia circumcincta* infection in sheep is one of the best understood of all host–parasite interactions, where detailed investigations have led to a much clearer understanding of the development of acquired immunity and the mechanisms involved in within-host regulation of parasite burden, length and fecundity [44–46]. Previous analyses show that there are two components to the host response in sheep. Immunity is acquired in response to exposure and develops in two stages, with lambs initially regulating worm growth and fecundity, and then worm number [47]. Immunoglobulin A (IgA) regulates worm growth and consequently fecundity as well as the numbers of eggs *in utero* [48–50]. The immunoglobulin E (IgE) response regulates larval establishment and therefore the number of worms in the host [44]. In addition, we have a detailed understanding of the genetic basis for variation in resistance to *T. circumcincta* infection, ranging from quantification of heritabilities to the identification of particular genes associated with resistance [46,51]. This detailed understanding of the epidemiology, immunology and genetics underpinning the sheep–*T. circumcincta* interaction makes it an ideal model system for the development of data-driven models, which capture and integrate information from these disciplines.

Here, we create an immunologically explicit model of infection dynamics in a sheep flock that links host genetic variation with variation in the two key immune responses described above to predict observed parasitological measures. One important advantage of this model is that by capturing the mechanistic link between the immune response and parasitological variables the model allows identification of improved markers for diagnosis and control. We first fit our model to genetic, immunological and parasitological data using approximate Bayesian computation (ABC). Second, using the fitted model, we contrast FEC with an immune marker (plasma IgA) as a measure of host resistance by comparing selective breeding in which selection is based either on low FECs or on high plasma IgA activity.

## 2. Model outline

### 2.1. Overview of the sheep–*Teladorsagia circumcinta* system

**Figure 1.**
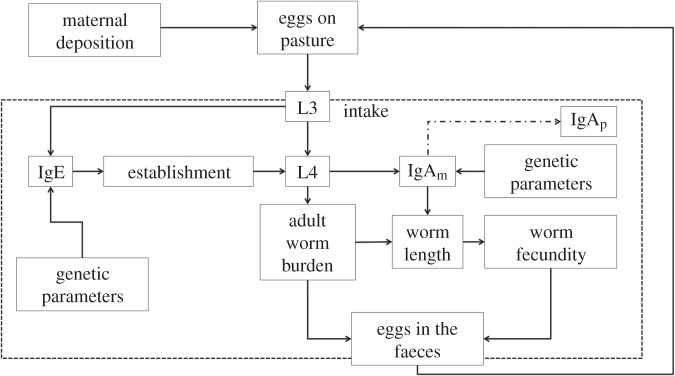
Model schematic. The region inside the dotted line represents the life cycle within the host. Worms develop from egg to adults with the larval stages L3 and L4 being explicitly included in the model. The L3 and L4 larval stages each influence a different component of the immune response of the host and, at the same time, different genetic parameters control the intensity of the immune response resulting from exposure to L3 and L4. The number of adults, as well as IgA, affects the average worm length, which is the major determinant of worm fecundity. The number of worms and the average fecundity determine the number of eggs excreted in the faeces each day. This deposition adds to the current pasture contamination. Arrows indicate the direction of the effect.

*Teladorsagia circumcincta* is a parasitic nematode that lives and reproduces as an adult in the abomasum (fourth stomach) of sheep. The worms lay eggs that are excreted with faeces onto pasture. The eggs hatch and after two larval stages (L1 and L2), they develop into infective L3 (stage 3 larvae). The L3 cannot develop further unless ingested by a potential host. Once inside the host, if they successfully establish, they moult to become L4 (stage 4 larvae) and subsequently progress to the adult stage (figure 1).

The life cycle of the nematode inside and outside the host is reproduced in our model, which is based on a published model of immunity to *T. circumcincta* infection in lambs [52]. Here, we modify and extend this model to capture infection dynamics in a flock of sheep in which genetic variation between individuals underpins heterogeneous immune responses to infection. A complete description of this individual-based discrete time (daily time step) model is given in the electronic supplementary material, but the key details that define the integration of the immunology and genetics are outlined below.

An important feature that distinguishes our model from previous models is that we explicitly model the protective mechanisms. Sheep control *T. circumcincta* infection through two key protective immune responses, which vary between hosts according to their genetic predisposition (figure 1). Antibodies including IgGI, IgA and IgE are produced against all parasitic stages of gastrointestinal nematodes, but protection is most strongly associated with IgE activity against L3 and IgA activity against L4. Larval establishment is controlled by the local IgE response, whereas worm fecundity is controlled by local IgA. As these two immune responses act upon different stages of the nematode life cycle, the L3 and L4 stages as well as the adults are modelled explicitly. The ingestion of L3 triggers mast cell degranulation that prevents worms from establishing [44]. L3 that establish develop into L4 and IgA responses to L4, possibly in conjunction with eosinophils [50], influence worm size and consequently worm fecundity [53]. We modelled IgA at two sites in the host: mucosal IgA (IgA_m_), which is unobserved (other than at post-mortem) and represents local IgA at the site of infection and affects worm length; and plasma IgA (IgA_p_), which represents the IgA that has migrated to the plasma and can be routinely measured in the bloodstream of live animals.

Worm fecundity is strongly correlated with the size of the worm; at the same time, worm size depends on the strength of the IgA response (specifically, IgA_m_) and a density-dependent effect of the number of worms in the animal [44]. Worm number and worm fecundity determine the egg deposition onto pasture and subsequently the number of infective L3 larvae available to be ingested.

Infective larvae are ingested during grazing. The amount of herbage consumed depends upon the size of animal. The growth of an animal during the course of the grazing season in our flock has been described in a standard manner by the Gompertz equation [52]. Rather than model herbage intake and larval intake separately, we modelled daily variation in larval intake among animals as a Poisson distribution with its parameter equal to the mean daily number of ingested L3 larvae. The mean number of ingested larvae increased concomitantly with lamb growth.

### 2.2. Genetic variation among lambs in immune responsiveness

Lambs differ in their capacity to mount the anti-establishment and anti-fecundity immune responses. This is captured by allowing parameters *ρ*_A_ and *ρ*_E_, which determine the rates at which IgA_m_ and IgE respond to parasite exposure of the lamb, to vary across the population. These parameters are assumed to be normally distributed across the flock and comprise an additive genetic component and an environmental component, as follows (with *i* representing each of the two immune responses):2.1
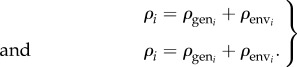
Total phenotypic variation is conventionally divided into additive genetic, non-additive genetic and environmental components [54]. As the non-additive component does not affect the response to selection, it was subsumed into the environmental component. The additive genetic and environmental components are sampled from normal distributions, which for immune response *i* can be written in the general form2.2a
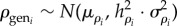
and2.2b
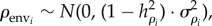
such that the additive genetic component has mean *μ*_*ρi*_ and the variance is partitioned between the genetic and the environmental component; 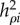 denotes the heritability of the trait, i.e. the proportion of the variance attributable to additive genetic effects [54]. These six parameters (*μ*_*ρ*A_, *μ*_*ρ*E_, 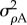, 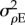, 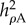, 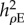) parametrizing the IgE- and IgA_m_-mediated immune responses are the free parameters used to fit the model to the field data. The other parameters in the model have been described in the literature and are assigned appropriate values (see the electronic supplementary material for details).

These parameters link host genetic variation with variation across the population in response to infection and ultimately determine the observed parasitological variables. As discussed above, one component of the immune response is related to the generation of mucosal IgA (IgA_m_), and is assumed to increase with rate *ρ*_A_ in proportion to the number of established L4 larvae, with a delay between exposure and initiation of an immune response of *z* days, and a half-life of *τ* days2.3
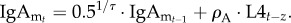
The fecundity of worms is defined as the number of eggs produced per adult female worm per day. It depends on worm length, which is determined by both worm burden (number) and IgA activity [44] as follows:2.4
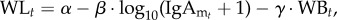
where *α* is the intercept term in the regression model, giving the expected mean length of adult worms in the absence of the immune response and density-dependent effects. *β* and *γ* are the coefficients for the effect of the immune response and worm number, respectively [44].

The number of eggs per worm on day *t,* Wf*_t_*, as a function of worm length, was adapted from the published relationship [55]2.5
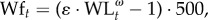
where the scaling by 500 accounts for the average weight of faeces (in grams) produced by lambs in this experiment to produce a fecundity in terms of eggs per worm per day.

The second component of the immune response controls the establishment of adult nematodes, which is strongly associated with mast cell degranulation and IgE activity [44]. The combined effect of these two responses we refer to as the establishment control factor (ECF). This is assumed to increase with rate *ρ*_E_ in proportion to the daily number of ingested L3 larvae, with a delay between exposure and initiation of an immune response of *z* days, and a half-life *τ* (measured in days):2.6
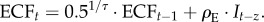
Under the assumption that establishment decreases over the grazing season as the immune system develops, we specified an establishment equation that reproduces field observations [52] summarized in a meta-analysis [56]. Establishment at time *t* is given by2.7
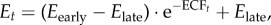
where *E*_early_ is the establishment in naive lambs, whereas *E*_late_ is the minimum establishment.

### 2.3. Observation processes

The FEC is a measure of the number of eggs in 1 g of faeces. The McMaster technique counts the number of eggs in 1/50th of a gram of faeces and is multiplied by 50. Measurement error was simulated by assuming Poisson counting error and accounting for the scaling up by a factor of 50. The model could therefore be used to output both the true FEC (i.e. without measurement error) and the predicted count with measurement error. Model fitting and selection were based on the measured rather than the true FEC.

Plasma IgA, denoted IgA_p_, has been previously shown to depend on IgA_m_ and the worm burden, WB, which is the number of worms at the site of infection [57]. We found a slightly improved fit to the data [58] with the following function relating IgA_m_ and worm biomass (WM, the product of the worm number and the mean worm length), with IgA_p_:2.8
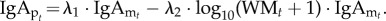
The IgA in the mucus is acting against the parasites, whereas the IgA in the plasma is a ‘spillover’ [58]. The advantage of modelling them separately is that IgA_p_ can be measured in live animals. We modelled IgE based on the meta-analysis of Gaba *et al.* [56]. Most local IgE is bound on the surface of mast cells, and the relationship between plasma IgE and local IgE is not known. Therefore, we did not attempt to model plasma IgE.

## 3. Selective breeding with alternative markers

The model can be used to compare different methods of parasite control such as grazing management, vaccination, nutritional supplementation and selective breeding, but here we choose to focus on selective breeding [59,60]. Currently, FECs are the marker most widely used to assess the intensity and severity of gastrointestinal nematode infection, and these are also used in selective breeding schemes. However, they are not particularly useful for *T. circumcincta* infections*,* because density-dependent constraints on fecundity mean that heavily infected animals produce few eggs [61]. The use of FECs is therefore hampered by their nonlinear and indirect relationship with host immune responses, and compounded by difficulties in obtaining accurate measurements of them. A possible alternative is IgA, which affects worm size and fecundity [53,62]. Here, we focus on a selection scheme for reduced FECs and compare it with selection for high plasma IgA activity to see which of these markers gives a better overall reduction in the intensity of infection.

### 3.1. Reference scenario

Our reference scenario is two selection schemes (selection on low FECs versus selection on high plasma IgA activity) run for 10 successive generations. For each year of selection, the model was used to simulate infection dynamics over the course of the grazing season, which started in early May and ended in September. The simulations ran for 140 days, updating daily, with simulated anthelmintic treatment every 28 days to match the timing of treatments that were administered to the animals in the field. We assumed a 100% effectiveness of the treatment, i.e. all adult and larval stages in the host of all gastrointestinal nematode species were killed. The model was based on data from a naturally infected flock [32]. This flock was treated with albendazole sulfoxide every 28 days from 4 to 24 weeks of age. FEC reduction tests were used every year to test drug efficacy, and there was no evidence for resistance during the trial. As the model is stochastic, 100 repeats were run, and the model outputs are taken to be the arithmetic means of the 100 repeats.

The initial flock in each repeat run of the model (i.e. generation 0) comprised 500 male and 500 female sheep with ages uniformly distributed between 1 and 3 years of age. The 500 female sheep were used to breed the next generation of 1000 lambs (500 male and 500 female), and were kept as a breeding flock of ewes that was updated every generation. As is common practice in sheep breeding, these ewes were not selected on performance. Each year, around one-third of the ewes are assumed to leave the flock owing to sale or mortality and replacement female sheep were picked at random from the flock of young sheep in that generation (1 year of age).

Each year, 25 males were used for breeding. To avoid inbreeding, rams are often bought in from outside and are chosen to improve the flock. We therefore assumed that each year the rams were unrelated to the ewes and conservatively assumed they had a distribution of resistance to infection similar to the current flock. In practice, in selective breeding, farmers would buy rams from more resistant flocks. These rams were used to breed the first generation. In subsequent years of selective breeding, the rams used mimicked the distribution of resistance among the best male lambs in the existing flock. Rams were selected for either low FECs or for high plasma IgA responses, with the 25 best rams selected for breeding. If more than 25 rams had a zero FEC, then 25 rams were chosen at random from this group. Each ram was mated to 20 ewes, resulting in each case in a twin male–female birth (1000 lambs in total).

### 3.2. Defining offspring parameters

To create a new generation of lambs, values for *ρ*_A_ (used in equation (2.3)) and *ρ*_E_ (used in equation (2.6)) for each new lamb were calculated. The additive genetic component (or breeding value) for each offspring is given by3.1
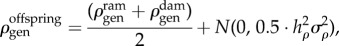
i.e. it is simply the mean of the parental values plus a Mendelian sampling term [63]. The environmental component is as given by equation (2.2).

### 3.3. Heritabilities

The heritabilities in the model were obtained by breeding one unselected generation of lambs and recording the parental values for each lamb. The heritability for a particular trait was then calculated by taking the ratio of the covariance of the parental mean and offspring values to the variance in the parental values:3.2
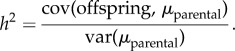
As heritability calculations are typically based on normal distributions [63], simulated values such as plasma IgA and worm length were normalized using a Box–Cox transformation [64] before calculating the covariances and variances.

### 3.4. The carryover effect

At the start of the season, the number of infective larvae on the pasture is largely determined by the deposition of worm eggs onto pasture by the ewes. As the flock improves through successive generations of selective breeding, the deposition by the replacement ewes will be less owing to their increased resistance. This relative reduction in start of season deposition is assumed to reflect the relative reduction in average FEC. The deposition, *S_y +_*_1_, for the following year therefore depends on the previous year's deposition, *S_y_*, via3.3
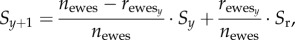
where *n*_ewes_ and *r*_ewes_ are the total and the replaced number of ewes, respectively, and *S*_r_ is the deposition of the replaced ewes which is calculated by scaling the initial deposition *S*_0_ with the reduction seen in the FECs (*S*_r_ = (FEC*_y_*/FEC_0_) · *S*_0_).

This carryover effect captures the expected reduction in the initial larval availability in subsequent generations as the flock becomes more resistant. Simulations were run with and without this effect but, unless stated otherwise, the results shown are for simulations with the carryover effect.

## 4. Field data and approximate Bayesian computation model fitting

The field data used to fit the model are generated from a study based on five cohorts, each of 200 lambs, from a naturally infected commercial flock in southwest Strathclyde [32,53]. The lambs were monitored monthly during their first grazing season (from mid-April to late September) for plasma IgA and FECs, and post-mortem analyses were performed late September and early October to obtain worm number and length. The parameters (*μ*_*ρ*A_, *μ*_*ρ*E_, 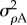, 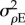, 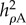, 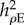), namely the means, variances and heritabilities of the immune response factors, are fitted to the field data. The values of all other model parameters have been extensively researched previously and were therefore determined from the literature as described in the electronic supplementary material. Six summary statistics from the field data were used as target values for the fitting (table 1); these values correspond to the average between the 5 years at the end of the grazing season (or post-mortem) and have been extensively analysed elsewhere [32]. The remaining field data were used to provide independent checks on the model fit (mean and variance of worm number).

**Table 1.** Summary statistics to be used as target model outputs taken from the fifth month of the grazing season for plasma IgA (IgA_p_) and faecal egg count (FEC), and at post-mortem (sixth month) for worm length (WL).

mean IgA_p_	0.2
mean log (FEC + 1)	1.85
variance of IgA_p_	0.027
variance of log (FEC + 1)	0.88
heritability (*h*^2^) of IgA_p_	0.56
heritability (*h*^2^) of WL	0.6

An ABC regression-based conditional density estimation algorithm was used to fit the model [65–67]. This assumes that we are conducting inference in a Bayesian framework where given a set of data *y* (i.e. the summary statistics in table 1), we seek to determine the posterior distribution *p*(*θ*|*y*) of the parameter vector *θ* given the data. In Bayesian inference, the posterior summarizes all information about the parameters conditional on the data and the specification of the model (including any fixed parameters) and the prior distribution of unknown parameters *p*(*θ*). A common approach we adopt here is that the prior assumes that the parameters are drawn from independent uniform distributions whose ranges are given in table 2.

**Table 2.** Ranges for the uniform prior distribution of the six parameters used to fit the model.

	*μ* (×10^−5^)	*σ*^2^ (×10^−11^)	*h*^2^
*ρ*_A_	1.7–2	8–10	0.5–1
*ρ*_E_	1.25–1.55	2.8–4.8	0.4–1

In the ABC algorithm, a so-called particle is defined as a set of values, one per parameter being fitted, so that each particle corresponds to a different value of the parameter vector *θ*. In our case, it contains the means and variances and heritabilities of the immune response factors *θ* = (*μ*_*ρ*A_, *μ*_*ρ*E_, 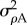, 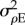, 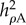, 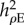). A different value for any one of the parameters in *θ* corresponds to a distinct particle. The steps are as follows.

(1) Given the unknown parameters *θ* and their prior distribution *p*(*θ*), *M* particles (*M* = 100 000 in our case) are generated by
  (a) drawing the parameter values randomly from the prior *p*(*θ*) for each particle (range of the uniform distributions in table 2) and
  (b) running the model for each particle.
(2) Compute the empirical standard deviation, across the *M* particles, for each of the simulated model outputs.
(3) Calculate the distance for each particle between the model and target outputs using the distance kernel as in Beaumont *et al.* [66].
(4) Choose a tolerance or proportion of points accepted; in our case, we accepted 1000 (1%) with the lowest distance.
(5) Weight the accepted particles as in Beaumont *et al.* [66].
(6) Correct the particles (i.e. adjust their position in parameter space) with the results from a weighted linear regression applied to the accepted particles as in Beaumont *et al.* [66].
(7) These corrected particles with the weights obtained in step 5 are taken to be random draws from an approximation to the posterior distribution *p*(*θ*|*y*).

**Figure 2.**
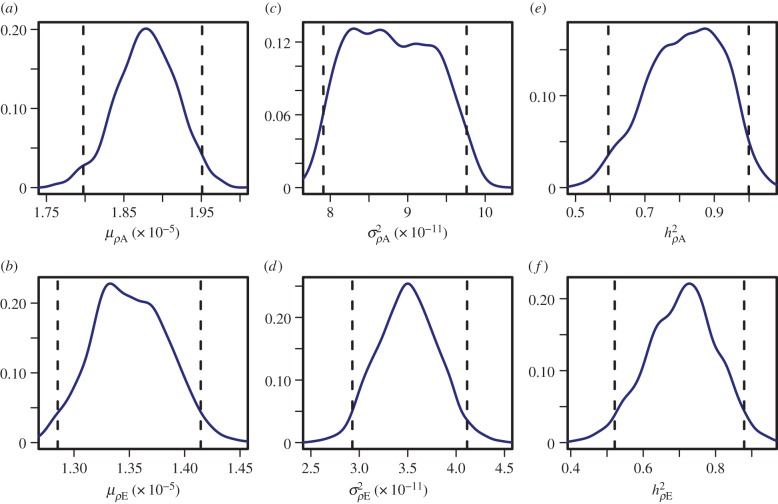
Approximate posterior distributions for the six fitted parameters: mean of *ρ*_A_ (*a*) and *ρ*_E_ (*b*), variance of *ρ*_A_ (*c*) and *ρ*_E_ (*d*), and heritability of *ρ*_A_ (*e*) and *ρ*_E_ (*f*). Vertical dashed lines indicate the 95% credible interval. (Online version in colour.)

This ABC algorithm yields an approximate posterior distribution for each of the fitted parameters (*μ*_*ρ*A_, *μ*_*ρ*E_, 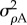, 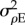, 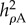, 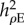; figure 2). Random draws from the corrected particles previously obtained are used in our model simulation. One draw is used for each of the repeats. The results presented in this paper are the average of 100 repeats.

### 4.1. Assessing model fit

To assess the fit of the model and observe the distribution of FEC and plasma IgA across the flock, we pooled the results of each repeat and averaged across the 100 repeats.

**Figure 3.**
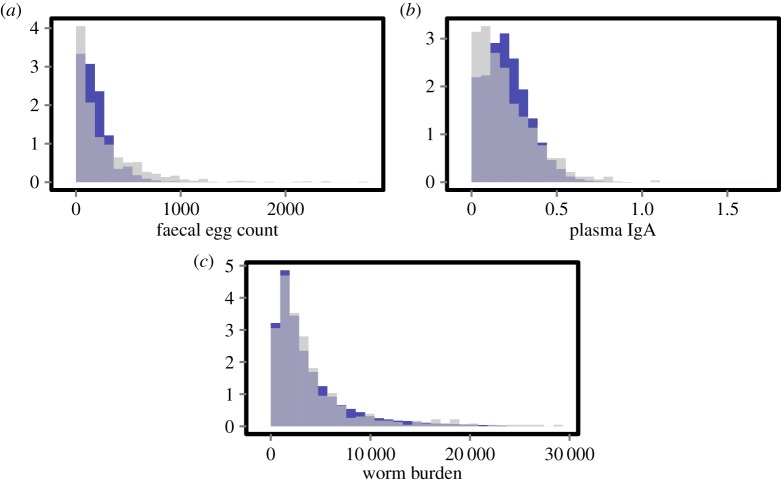
Comparison between field observations (light) and simulated values (dark) of (*a*) faecal egg counts, (*b*) plasma IgA and (*c*) worm burden (number). Intermediate colour is the overlap. To generate these distributions across the flock, we ran the model once with a large number of animals (100 000), using the ‘best fit particle’ (i.e. the particle with the smallest distance based on the ABC distance kernel). (Online version in colour.)

The model, as expected after the fitting, successfully reproduces the mean and the variance of the FEC and plasma IgA. Moreover, the distributions for each of these observed quantities are also successfully reproduced (figure 3*a,b*). We also investigated the model fit to quantities the model was deliberately not fitted to. For the worm number, the estimated mean, variance and distribution obtained in the model are also similar to the field observations (figure 3*c*). The heritability of the FECs was also calculated (as in equation (3.2); value obtained 0.22) and although the model was not fitted to it, is in accordance with the field observation (0.2–0.3). These outcomes provide additional independent validation of the model.

## 5. Results

**Figure 4.**
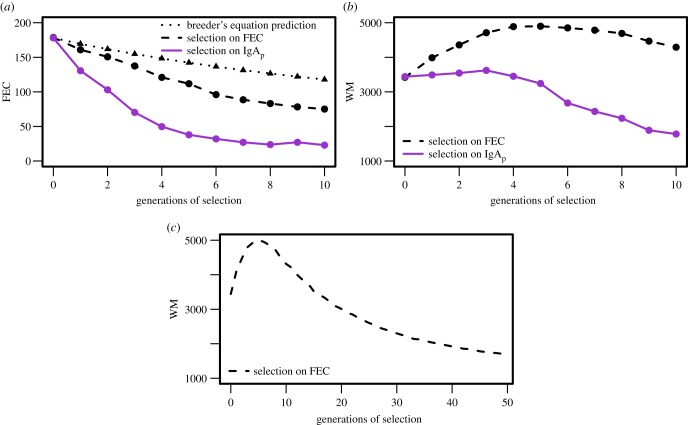
(*a*) Mean flock faecal egg count (FEC) at the end of the grazing season over 10 generations of selection. The dotted line is the predicted response using the breeder's equation; the dashed dark line shows the response to selection based on low FECs; the solid light line shows the response to selection based on high plasma IgA activity. (*b*) Mean flock worm biomass (WM) at the end of the grazing season over 10 generations of selection: selection based on low FEC in dashed dark; selection based on high plasma IgA activity in solid light. (*c*) Average flock WM at the end of the season for 50 generations of selection on low FEC. (Online version in colour.)

In our reference scenario, we compared model predictions for selective breeding based on low FECs versus high plasma IgA. We also included the breeder's equation prediction (figure 4*a*), which is the expected response to selection estimated from the average difference between the whole parental generation and the subset of selected parents [54]. In our case, it is the difference in average FEC breeding values between all the male lambs and the subset of 25 selected rams.

Under each selection scenario, the mean FEC across the flock is calculated at the end of each grazing season. The reduction in FEC at the end of each grazing season based on selection for low FEC was 1.7 times faster than is estimated by the breeder's equation over 10 generations (figure 4*a*, dotted line). A more rapid decrease in the mean flock FEC is observed under selection for high plasma IgA responses (figure 4*a*, light solid line). By the seventh generation, selection on plasma IgA achieved a drop in FEC of almost 85%, whereas selection based on FEC achieved a reduction of approximately 50%.

We defined the WM to be the product of worm number and average worm length [58]. As this quantity accounts for the reported decrease in worm activity and fecundity in shorter worms [44], we use this as a measure of the intensity and pathology of infection. WM decreases by almost half after 10 generations of selection based on high plasma IgA activity while, when selecting on low FECs, the WM slightly increases before starting to drop (figure 4*b*).

Under selection for low FECs, after the initial increase in WM, running simulations for more than 10 generations shows that values for WM similar to those prior to selection are obtained after 15 generations of selection. However, it takes 50 generations of selection based on low FEC to obtain similar values of WM to the ones obtained after only 10 generations of selection on plasma IgA (figure 4*c* in comparison with figure 4*b*, light solid line).

## 6. Discussion

This paper presents an immunologically explicit model of an important host–parasite system and links host genetic variation with variation in the two key immune responses, accurately reproducing the means and distributions of parasitological and immunological observations. To the best of our knowledge, this is the first data-driven model of the host–nematode interaction that combines the epidemiology, the genetics and the explicit development of the adaptive immune response. This model therefore represents an important step forward in host–parasite modelling and moreover, provides a tool that can be used for multiple purposes. In this paper, we focused on selective breeding schemes as a means of parasite control and tested novel markers for the efficient identification of resistant animals.

An important advantage of our model is that we connect the underlying genetic variation with variation in the two protective components of the immune response across the host population to predict observed parasitological variables. By capturing the mechanistic link between the immune response and parasitological variables, the model allows us to identify alternative markers for diagnosis and control. Novel markers could offer substantial improvements over the widely used FEC, which suffers from substantial measurement error, and is only indirectly and nonlinearly related to the host immune response. Specifically, we hypothesized that the IgA response would provide a better marker than FEC, because IgA activity directly affects worm length and fecundity and therefore FEC, but is subjected to less observation error than the FEC. To test this hypothesis, we compared the outcome of selection schemes based on selection for low FEC versus selection for high plasma IgA activity.

The estimated response to selection based on low FECs is much faster than that predicted by the breeder's equation, which is typically used to predict the response to selection for quantitative traits when there is no change in the environment during selection. Our result is similar to the one presented in Bishop & Stear [27], although their predicted end of season average FEC was much higher as the flock was initiated with a higher mean infection load. Our predictions are consistent with independent field observations; Karlsson & Greeff [68] calculated in their Rylington Merino flock a genetic reduction of 2.7% in FECs per year in their selection scheme based on both production traits and FECs. In our model, the predicted response rate was a comparable average reduction in FECs of 4.2% per year for selection based solely on FEC.

We have shown that an immune marker, plasma IgA, which can be sampled in live animals, provides a potentially valuable alternative to FECs. However, the ultimate objective of a selection scheme is to reduce the pathology associated with infection. To this end, we defined WM as the product of worm length and worm number; because small worms are thought to be less damaging than large ones [69], this measure provides a better measure of the pathology associated with infection than worm number alone. Thus, the outcome of the selection scheme should be assessed not only in terms of FEC, but also in terms of the predicted reduction in WM.

Our comparisons between selection schemes based on low FEC versus high plasma IgA activity show that after a few years of stabilization, the worm mass decreases in both selection scenarios. Selection on low FEC will indirectly act on both components of the immune response, reducing the establishment, and thus the worm number, and the fecundity. With a lower establishment, the number of L4 will also be smaller, which in turn causes animals to have a weaker anti-fecundity response. For the first years of selection, the reduction in adult worms (owing to the reduction in establishment) is not enough to compensate for a slightly higher mean worm length (owing to the weaker anti-fecundity response), which causes the overall WM to be higher.

Although both selection schemes successfully reduce worm mass in the long run, selection for high plasma IgA reduces WM substantially more quickly, with a decrease of around 50% in 10 generations. Although it is commonly assumed that selection directly on a trait is the most effective way to alter it, our system differs: because plasma IgA has a higher heritability than FECs and high levels of IgA reduce worm growth and fecundity, selection on this trait reduces both the egg output and WM more quickly than direct selection on FEC.

Our model uses monthly anthelmintic treatment. This is a widely used method of parasite control particularly when pasture contamination is high. Our model was validated by testing it against field data from a farm that treated lambs every 28 days. Other farmers treat less frequently or use anthelmintics that are less efficacious because of drug resistance in the parasite population. These scenarios could lead to higher levels of infection and stronger immune responses depending on the initial pasture contamination. However, there are too few detailed field studies to predict the consequences with confidence.

Future models will examine the impact of selection on growth as a production trait. This will allow us to evaluate IgE as a marker of resistance. The IgE-mediated hypersensitivity response is associated with reduced larval establishment [44] but is weakly associated with reduced growth [70]. Binding of parasite molecules to IgE induces mast cell degranulation which breaks down the tight junction between epithelial cells and induces a relative protein deficiency [3]. Therefore, IgE is less attractive as a marker than IgA, which is not associated with reduced growth rate [3]. In future work, we will extend the model to allow growth to depend on worm number and IgE activity. We will then be able to contrast selection schemes that use growth, IgA and IgE to identify the optimal combination of markers.

Our model addresses long-standing gaps and issues in host–parasite models, simultaneously capturing aggregation of infection burdens, explicitly modelling the development of the adaptive immune response and the role of host heterogeneity. This step forward has been facilitated by our understanding of immunological mechanisms of control, extensive parasitological and immunological observations, and the availability of pedigree data to determine the heritability of these traits. Fitting these data to a mechanistic model has enabled us to characterize the variation and heritability of the underlying immune responsiveness, providing new insights into the role of host heterogeneity in the host–parasite interaction. The most promising methods of control in parasite infections of livestock—selective breeding, improved nutrition, vaccination—all involve improving the immune response. This model provides not only a deeper understanding of the role of host heterogeneity and adaptive immunity, but also a valuable tool for improved understanding, analysis and prediction of the impacts of a wide range of control measures.

In conclusion, this paper has presented a model of developing immunity through the grazing season and has been applied, as an example, to the comparison of selection schemes that use different indicators of resistance. The model is immunologically and genetically explicit, and it has been fitted to field observations. The results show that IgA can be a better indicator of resistance to infection than FEC and that selection schemes based on parasite-specific IgA activity are likely to be more effective than selection based on FEC.

## Supplementary Material

Full Model Description
