# Supplementary material for: An explicit immunogenetic model of gastrointestinal nematode infection in sheep
Source: J R Soc Interface. 2014 Oct 6;11(99):20140416. doi: 10.1098/rsif.2014.0416 (PMC4233724; doi:10.1098/rsif.2014.0416)
Supplement: Full Model Description [file rsif20140416supp1.pdf]

Supplementary Material – Full Model description

The model is based on a previous model of immunity to *T. circumcincta* infection in lambs [1] which we extend here to capture individual variation in response to infection across a flock of sheep. We first present the model equations, and then discuss model parameterisation.

Nematode life cycle

We base this component of the model, with certain modifications detailed below, on Bishop and Stear [2] which captured the nematode life cycle with two equations, one for the worm burden and one for infective larvae in the field (L3 larvae). We have modified these equations to additionally explicitly include the L4 larvae, which trigger the anti-fecundity response.

The number of adult worms (WB) per lamb on day *t*, WB_t, depends on the worm burden on day *t-1*; the mortality rate of the L4 larvae and adult worms, m_{L4} and m_A, respectively; the number of larvae ingested *j* days ago, I_{t-j}; and the proportion of ingested L3 larvae that establish in the host and progress to become L4 larvae, E_{t-jL4} (equation S1). The pre-patent period, $j = j_{L3} + j_{L4}$, is the time taken for ingested L3 larvae to develop to a fully-grown adult worm, where j_{L3} and j_{L4} are the times, post ingestion, spent in the L3 and L4 stages respectively.

$$WB_t = WB_{t-1}(1-m_A) + I_{t-j}E_{t-j_{L4}}(1-m_{L4})^{j_{L4}} \qquad \text{S1}$$

The infective larvae on pasture on day *t*, L_t, depends on the infective larvae present on day *t-1*; the number of ingested larvae, I_{t-1}; the mortality rate of infective larvae, m_{L3}; the number of eggs deposited on pasture by ewes, S_{t-u}; the fecundity Wf_{t-u} and worm burden, WB_{t-u}, each *u* days earlier, where u_t is the number of days taken to develop from egg to infective stage (here, assumed to vary monthly); and the proportion of eggs reaching the infective L3 stage (equation S2) at day t, e_t, as follows:

$$L_t = \left(L_{t-1} - I_{t-1}\right)\cdot(1-m_{L3}) + \left(S_{t-u_t} + WB_{t-u_t}Wf_{t-u_t}\right)\cdot e_t \qquad \text{S2}$$

The number of L4 in a given animal on day *t*, $L4_t$, depends on the rate at which L4 die, the rate at which ingested larvae (L3) become L4 and the rate at L4 become adults and is given by

$$L4_t = L4_{t-1}(1-m_{L4}) + I_{t-j_{L3}}E_t - I_{t-j}E_{t-j_{L4}}(1-m_{L4})^{j_{L4}} \quad \text{S3}$$

where m_{L4} is the mortality rate for the L4 stage, $I_{t-j_{L3}} \cdot E_t$ is the number of larvae ingested j_{L3} days previously establishing to become L4, and $I_{t-j} \cdot E_{t-j_{L4}}$ scaled by the mortality term gives the number of ingested and established larvae that are still alive and ready to leave the L4 stage to become adults.

Immune Responses

IgA activity against L4 is strongly associated with reduced parasite fecundity [3]. We have modelled both mucosal and plasma IgA because mucosal IgA acts at the site of infection, whereas the related quantity, plasma IgA, is the quantity measured in the blood stream. Mucosal IgA, denoted IgA_m, is produced in response to the L4 population, and is assumed to increase with rate ρ_A in proportion to the number of L4, with a delay from exposure to initiation of an immune response of z days and a half-life of τ days, as follows:

$$IgA_{m_t} = 0.5^{1/\tau} \cdot IgA_{m_{t-1}} + \rho_A \cdot L4_{t-z} \quad \text{S4}$$

Plasma IgA, denoted IgA_p, has been previously shown to depend on IgA_m and the worm burden, *WB*, at the site of infection [4]. We found an improved fit to the data [5] to be given by a relationship between worm biomass, *WM*, which is the product of the worm burden and the mean worm length, IgA_m and IgA_p as follows:

$$IgA_{p_t} = \lambda_1 IgA_{m_t} - \lambda_2 \log_{10}\left(WM_t + 1\right) IgA_{m_t} \quad \text{S5}$$

The establishment of adult nematodes is strongly associated with mast cell degranulation and IgE activity [3], whose effects we capture jointly via establishment control factor (ECF). This is assumed to increase with rate ρ_E in proportion to the number of ingested L3 larvae and is assumed to decay with a half-life of τ days, as follows:

$$ECF_t = 0.5^{1/\tau} \cdot ECF_{t-1} + \rho_E \cdot I_{t-z} \quad \text{S6}$$

To capture the decrease in establishment over the grazing season as the immune system develops, we created an establishment equation that reproduces field observations that were summarised in a meta-analysis [6]. The establishment, E_t, at time, t, is expressed in terms of E_{early}, the parasite establishment for naïve lambs, and E_{late} which is the minimum long term establishment, as follows:

$$E_t = \left(E_{early} - E_{late}\right) \cdot e^{-ECF_t} + E_{late} \quad \text{S7}$$

Nematode fecundity

The fecundity of worms depends on worm length, which is known to be influenced by both worm burden and IgA activity [3]. A regression model fitted to the data gave the following relationship (equation S8) between worm length, WL, mucosal IgA activity and worm burden

$$WL_t = \alpha - \beta \cdot log_{10}\left(IgA_{m_t} + 1\right) - \gamma \cdot WB_t \quad \text{S8}$$

where α is the intercept term in the regression model, giving the expected mean length of adult worms in absence of the immune response and density dependent effects. β and γ are the coefficients for the effect of the immune response and worm burden respectively [3].

The numbers of eggs per worm on day *t*, Wf_t, was taken from the published relationship [7]. Adult size ranged from 0.7cm to 1.2cm [3] and worms smaller than the threshold size (estimated to be 0.7cm) are assumed to not reach maturity and therefore produce no eggs. The fitted relationship is given by

$$Wf_t = (\varepsilon WL_t^{\omega} - 1) \cdot 500 \quad \text{S9}$$

where the scaling by 500 accounts for the average weight of faeces (in grams) produced by lambs in this experiment to produce a fecundity in terms of eggs per worm per day.

Ingestion and egg deposition in the field

We assume that the number of larvae ingested by lambs and their faecal deposition onto pasture depends on the food consumption and therefore, the weight of the animal. Lamb weights were assumed to follow a Gompertz equation [1]

$$weight_t = \theta \exp\left[\mu\left(1 - e^{-\kappa t}\right) / \kappa\right] + \phi \quad \text{S10}$$

where Φ is the weight at birth and the parameters θ, μ, κ were estimated by fitted the expression to observed lamb weights [1].

The herbage consumed per lamb per day, Q_t, is assumed to be proportional to the weight gain in the lamb since birth [1]

$$Q_t = \nu(weight_t - \phi) \quad \text{S11}$$

The number of infective larvae ingested per lamb depends on the larvae available on pasture, L, and the herbage consumed, Q, is proportional to the stocking density of lambs on pasture, D, and inversely proportional to herbage density, H, and is given by

$$I_t = L_t Q_t \left(\frac{D}{H}\right) \quad \text{S12}$$

Using the growth curve given by equation (S10), we can calculate the faecal deposition in pasture and the quantity of egg deposited as follows

$$FEC_t = \frac{WB_t \cdot Wf_t}{weight_t \cdot f} \qquad \text{S13}$$

where the numerator is the total number of eggs in faeces per day whilst the denominator is the mass of faeces produced in a day, assumed to be proportional to the size of the animal.

Model parameterisation

Table S**1** gives the variables used along with the equations that govern their dynamics.

	Variable	Equation
**WB**	Worm burden	S1
**L**	L3 population on pasture	S2
**L4**	L4 burden	S3
**IgAm**	Immune response – Mucosal IgA (site of infection)	S4
**IgAp**	Immune response – Plasma IgA (blood)	S5
**ECF**	Immune response - Establishment control factor	S6
**E**	Worm establishment	S7
**WL**	Worm length	S8
**Wf**	Worm fecundity	S9
weight	Lamb weight	S10
**Q**	Daily herbage intake	S11
**I**	Daily ingestion of infective larvae	S12
**FEC**	Faecal egg counts	S13

Table S1. Model variables and the respective equations that govern their dynamics

Table S**2** gives the parameter values used in the model either with a reference from the literature, or with a justification for the selected values for parameters not estimated in the literature, or where estimates vary.

	Parameter	Value	Reference
e_t	Larval development success in the field (per day)	0.09(1+sin(πt/140))	(see below for justification)
m_{L3}	Mortality rate for L3 in the field (per day)	0·008	[8]
m_{L4}	Mortality rate for L4 (per day)	0·01	(see below for justification)
m_A	Mortality rate for adults (per day)	0·0307	[9]
u_t	Development time from egg to L3 (days)	6-12	(see below for justification)
j_{L3}	Time from ingestion to L4 stage (days)	2	[10]
j_{L4}	Time from L4 to adult stage (days)	14	[10]
L_0	Initial larval availability (larvae per lamb)	10 000	[1]
S_t	Ewes egg deposition (eggs per lamb)	Initially 250 000 with linear decrease to 0 at day 84	[1]
f	Faeces per Kg of bodyweight (g)	20	[1]
D	Stocking density (lambs/ha)	35	[11]
H	Herbage density (Kg DM/ha)	1200	[11]
τ	Half-life of antibodies (days)	8·1	[12]
z	Lag in acquisition of immunity (days)	7	[1]
ρ_A	IgA response factor	See equation 2.1-2.2	
ρ_E	Establishment response factor	See equation 2.1-2.2	

Table S2. Model parameters plus the value used in the model and the reference (where available).

Mortality rates depend on the larval stage. The pre-infective larval stages are the most vulnerable to weather conditions and predation and consequently have the highest mortality rate; this mortality is captured in the larval development success term, e_t. The mortality of L4 is not available in the literature, presumably because it would be difficult to measure experimentally. As the L4s are shielded in areas of the intestine known as gastric

pits while they develop, the mortality, m_{L4}, is believed to be low relatively to the adult mortality rate; therefore it is set at 0.01 per day.

The time taken to develop from egg to infective stage (L3), u, ranges from 6 days to 4 weeks in the literature [13-15], varying in response to environmental factors such as humidity and temperature and by nematode species [15]. Salih and Grainger [16] proposed the following equation for the development time of *T. circumcincta* in the UK as a function of temperature:

$$u_t = 132 \cdot T^{-1.1018} \qquad \text{S14}$$

Historic weather records for years 1992 to 1996 (years when our data were collected) were used to provide the average monthly temperatures for May to September (the period simulated by the model). Using S14 we calculated mean monthly development times (u_t), which ranged from 6 to 12 days. This variation in development time from egg to L3 coupled with the mortality in this pre-infective stage influence the success, e_t, which is calculated monthly as follows:

$$e_t = (1 - m_p)^{u_t} \qquad \text{S15}$$

where m_p is the mortality rate of the pre-infective stage (0.23, [13]) and u_t is the development time. To these monthly data (red dots, figure S1), using maximum likelihood, we fitted a sinusoidal curve to represent the daily change in larval success over the season (black dots, figure S1).

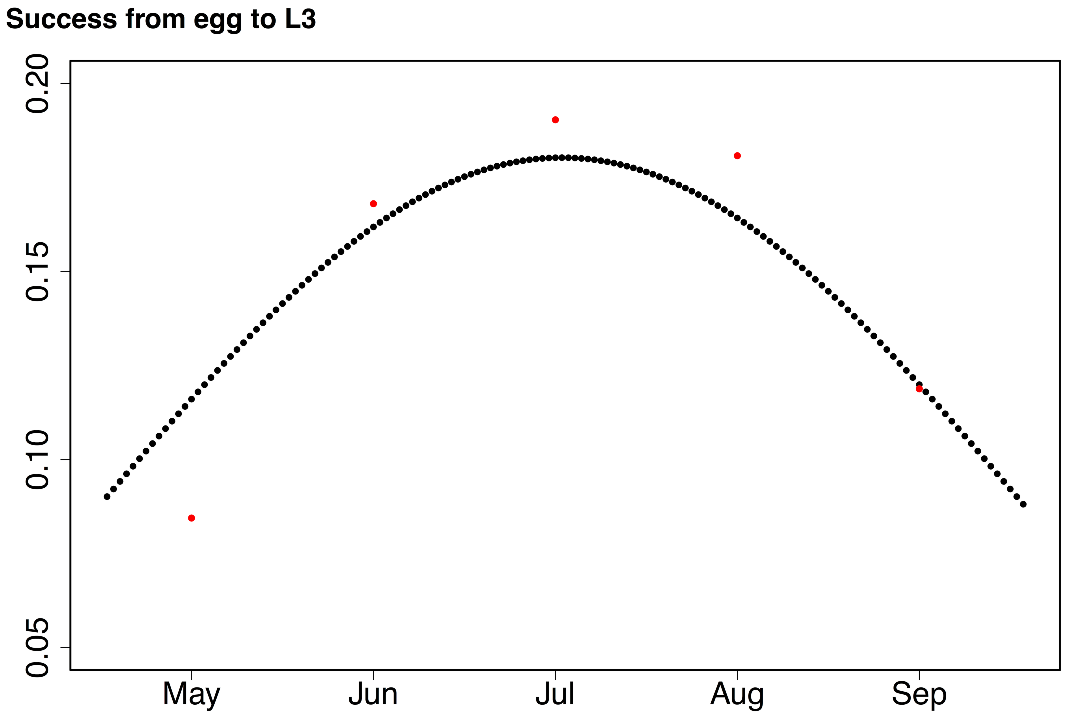

Figure S1. Proportion of eggs successfully developing into L3 larvae. The red dots are the data points (one for each month). Black dots are the fitted sinusoidal success curve used in the model for parameter e_t.

The time post ingestion taken to develop to the L4 stage, j_{L3}, lies between 1 to 3 days [10], which is the time taken to ex-sheath, travel to the abomasum and settle in a gastric pit and moult; the mean value of 2 days was chosen for our simulations.

The next development interval, the time taken by the L4 stage to develop into adults is particularly important because it is L4 antigens that trigger the IgA immune response. The first adults appear 12 days post ingestion while the last L4s become adults 20 days post ingestion [10]. A mean value of 16 days was chosen for the pre-patent period, with 14 days assigned to the development from L4 to adult, j_{L4}, (since we assigned 2 days to j_{L3}).

Model coefficients are listed in **Table S3**.

**Coefficient**	**Value**	**Related parameter**
α	1·071	
β	0·65	Worm length, l; [3]
γ	$5·2×10^{-6}$	
ε	1·12	Worm fecundity, n; [3]
ω	0·41	
θ	$3·6×10^{-5}$	
μ	0·614	Live weight, W; [1]
κ	0·0471	
ϕ	10·18	Live weight, W and herbage consumed, Q; [1]
ν	0·109	Herbage consumed, Q; [1]
E_{early}	0·4	Establishment, E; [1]
E_{late}	0·0	
$λ_1$	3·98	Transfer equation, IgA_p; [5]
$λ_2$	1·02	

Table S3. Coefficients used in the model equations with the relevant parameter.

References

1. Singleton D. R., Stear M. J. & Matthews L. 2011 A mechanistic model of developing immunity to Teladorsagia circumcincta infection in lambs. *Parasitology* **138** 322-32.

2. Bishop S. C. & Stear M. J. 1997 Modelling responses to selection for resistance to gastro-intestinal parasites in sheep. *Animal Science* **64**, 469-78.

3. Stear M. J., Bishop S. C., Doligalska M., Duncan J. L., Holmes P. H., Irvine J., McCririe L., McKellar Q. A., Sinski E. & Murray M. 1995 Regulation of egg production, worm burden, worm length and worm fecundity by host responses in sheep infected with Ostertagia circumcincta. *Parasite Immunology* **17**, 643-52.

4. Sinski E., Bairden K., Duncan J. L., Eisler M. C., Holmes P. H., McKellar Q. A., Murray M. & Stear M. J. 1995 Local and plasma antibody responses to the parasitic larval stages of the abomasal nematode Ostertagia circumcincta. *Vet Parasitol* **59**, 107-18.

5. Prada Jiménez de Cisneros J., Matthews L., Mair C., Stefan T. & Stear M. J. 2014 The transfer of IgA from mucus to plasma and the implications for diagnosis and control of nematode infections. *Parasitology*. (http://dx.doi.org/10.1017/S0031182013002321)

6. Gaba S., Gruner L. & Cabaret J. 2006 The establishment rate of a sheep nematode: revisiting classics using a meta-analysis of 87 experiments. *Vet Parasitol* **140**, 302-11.

7. Stear M. J. & Bishop S. C. 1999 The curvilinear relationship between worm length and fecundity of Teladorsagia circumcincta. . *Int J Parasitol* **29**, 777-80.

8. Gibson T. E. & Everett G. 1972 The ecology of the free-living stages of Ostertagia circumcincta. . *Parasitology* **64** 451-60.

9. Hong C., Michel J. F. & Lancaster M. B. 1986 Populations of Ostertagia circumcincta in lambs following a single infection. *Int J Parasitol* **16**, 63-7.

10. Denham D. A. 1969 The development of Ostertagia circumcincta in lambs. *Journal of Helminthology* **43**, 299-310.

11. Waller P. J., Dobson R. J., Donald A. D. & Thomas R. J. 1981 Populations of Strongyloid Nematode Infective Stages in Sheep Pastures - Comparison between Direct Pasture Sampling and Tracer Lambs as Estimators of Larval Abundance. *Int J Parasitol* **11**, 359-67. (Doi 10.1016/0020-7519(81)90006-0)

12. Henderson N. G. Immunity to Teladorsagia circumcincta infection in Scottish Blackface sheep: an investigation into the kinetics of the immune response, antigen recognition and the MHC: University of Glasgow; 2002.

13. Learmount J., Taylor M. A., Smith G. & Morgan C. 2006 A computer model to simulate control of parasitic gastroenteritis in sheep on UK farms. *Vet Parasitol* **142** 312-29.

14. Leathwick D. M., Barlow N. D. & Vlassoff A. 1992 A model for nematodiasis in New Zealand lambs. *Int J Parasitol* **22**, 789-99.

15. Leathwick D. M. 2013 The influence of temperature on the development and survival of the pre-infective free-living stages of nematode parasites of sheep. *N Z Vet J* **61**, 32-40. (10.1080/00480169.2012.712092)

16. Salih N. E. & Grainger J. N. R. 1982 The Effect of Constant and Changing Temperatures on the Development of the Eggs and Larvae of Ostertagia-Circumcincta. *J Therm Biol* **7**, 35-8. (Doi 10.1016/0306-4565(82)90017-1)
